# Quantifying the Varying Predictive Value of Physical Activity Measures Obtained from Wearable Accelerometers on All-Cause Mortality over Short to Medium Time Horizons in NHANES 2003–2006

**DOI:** 10.3390/s21010004

**Published:** 2020-12-22

**Authors:** Lucia Tabacu, Mark Ledbetter, Andrew Leroux, Ciprian Crainiceanu, Ekaterina Smirnova

**Affiliations:** 1 Department of Mathematics and Statistics, Old Dominion University, Norfolk, VA 23529, USA; 2 Department of Mathematics, University of Lynchburg, Lynchburg, VA 24501, USA; ledbetter_mk@lynchburg.edu; 3 Department of Biostatistics & Informatics, Colorado School of Public Health, University of Colorado, Aurora, CO 80045, USA; andrew.leroux@cuanschutz.edu; 4 Department of Biostatistics, Johns Hopkins University, Baltimore, MD 21205, USA; ccrainic@jhsph.edu; 5 Department of Biostatistics, Virginia Commonwealth University, Richmond, VA 23298, USA; ekaterina.smirnova@vcuhealth.org

**Keywords:** accelerometry, physical activity, logistic regression, mortality, prediction horizon, NHANES

## Abstract

Physical activity measures derived from wearable accelerometers have been shown to be highly predictive of all-cause mortality. Prediction models based on traditional risk factors and accelerometry-derived physical activity measures are developed for five time horizons. The data set contains 2978 study participants between 50 and 85 years old with an average of 13.08 years of follow-up in the NHANES 2003–2004 and 2005–2006. Univariate and multivariate logistic regression models were fit separately for five datasets for one- to five-year all-cause mortality as outcome (number of events 46, 94, 155, 218, and 297, respectively). In univariate models the total activity count (TAC) was ranked first in all five horizons (AUC between 0.831 and 0.774) while the active to sedentary transition probability (ASTP) was ranked second for one- to four-year mortality models and fourth for the five-year all-cause mortality model (AUC between 0.825 and 0.735). In multivariate models age and ASTP were significant in all one- to five-year all-cause mortality prediction models. Physical activity measures are consistently among the top predictors, even after adjusting for demographic and lifestyle variables. Physical activity measures are strong stand-alone predictors and substantially improve the prediction performance of models based on traditional risk factors.

## 1. Introduction

Physical activity (PA) measured using wearable accelerometry devices holds great promise for remote monitoring of physiological health and mortality risk assessment [1]. A growing number of health studies use objective PA measurements obtained from accelerometry to either complement or replace the traditional self-assessment questionnaires [2]. Recent studies have shown that objective physical activity measures are among the major mortality risk factors [3,4,5,6,7,8,9,10]. While some traditional risk factors, such as age, are not modifiable, physical activity can be intervened on, which could decrease an individual’s risk of mortality [11,12,13]. Moreover, higher PA is associated with better physical performance and a healthier and longer life [14,15,16,17]. Translating these findings into actionable public health guidelines and intervention monitoring requires a better understanding and quantification of the association between objectively measured physical activity in the free-living environment and the risk of mortality.

Physical activity has an important impact on an individual’s health and quality of life [18]. Epidemiological studies over the past 50 years have shown that decline in physical activity, which is most likely associated with a number of unfavorable changes in biochemical and physiological processes, is associated with cardiovascular and all-cause mortality [19]. There is extensive evidence that physical activity reduces the risk of developing many chronic diseases [20] including incident dementia [21,22,23], cardiovascular diseases [24,25,26], and diabetes [27,28,29,30]. Public health recommendations emphasize the importance of lifestyle changes to increase physical activity via common behaviors such as walking, climbing stairs, and doing housework [31,32]. However, many older adults remain predominantly sedentary [33,34,35], which could contribute to increased disability and even premature death.

To date, the National Health and Nutrition Examination Survey (NHANES) 2003–2004 and 2005–2006 [33] and the UK Biobank [36] are the largest open-source population-based studies that included physical activity measured by wearable accelerometry devices. Total time spent in light, moderate, and vigorous physical activity has been previously shown to be associated with mortality outcomes in the NHANES accelerometry data [1,3]. Fractal analysis of NHANES accelerometer signals further revealed the potential of actigraphy data to serve as a clinical biomarker for mortality risk [1]. Recently, Smirnova et al [9] studied the predictive performance of physical activity summary measures obtained from hip-worn accelerometer in the subset of 2978 NHANES 2003–2004 and 2005–2006 participants over the age of 50. Physical activity measures were among the strongest five-year all-cause mortality predictors in logistic regression models. However, single horizon models could miss important time-specific effects of physical activity and other traditional measures of predictive performance. In the UK Biobank data Leroux et al [37] showed that similar physical activity measures obtained from wrist-worn accelerometers were among the top mortality predictors in Cox regression models. However, previous analyses have not explicitly considered the time-dependent predictive value of PA variables. In summary, previous reports do not explore the individual and joint predictive performance of objective measures of physical activity as a function of mortality prediction horizon. Thus, there is current need for conducting analyses for multiple prediction horizons to provide complementary information to previous studies that have either focused on one-time horizon [9] or on time-to-event analyses [37].

In this study, we examine the predictive performance of physical activity derived measures in one- to five-year all-cause mortality for 2978 individuals over the age of 50 in the NHANES 2003–2006 study. We hypothesize that: (1) physical activity features are highly predictive of all-cause mortality in both short (one to two years) and medium (three to five years) follow up periods; and (2) the predictive value of PA features is most strong over shorter follow-up periods, but persists when looking at longer follow-up times. To test these hypotheses, the objectives of the current study are to: (1) identify the strongest horizon-specific mortality predictors in individual and combined models; (2) develop a combined prediction model for one- to five-year all-cause mortality; and (3) examine the changes in the strength of mortality predictors as a function of prediction horizon.

## 2. Materials and Methods

### 2.1. Study Population

The NHANES is a large study conducted by the Centers for Disease Control (CDC) to assess the health and nutritional status of the US population [38]. The study collects information on the demographic, socioeconomic and health status of the US population using survey questionnaires and physical activity data using hip accelerometers. The NHANES 2003–2004 and 2005–2006 data were downloaded, processed, and combined with survey weights and mortality data (updated through 2015) using the R package rnhanesdata [39]. Under specific assumptions on the missing data patterns the function reweight accel() in the rnhanesdata package was used to recalculate the sampling weights for the selected subset of participants.

The NHANES 2003–2004 and 2005–2006 have a total of 14, 631 study participants with accelerometry records. For the purpose of our analyses some study participants were excluded for the following reasons: (1) were younger than age 50 and older than 85 years at the time of accelerometers wear (10,859 participants); (2) had missing BMI and education information (41 participants); (3) had missing systolic blood pressure, total or HDL cholesterol measurements (293 participants); (4) had missing mortality information (21 participants); (5) had fewer than 3 days of activity data with at least 10 h of estimated wear time or had various data reliability issues (517 participants). Non-wear time was estimated as intervals with at least 90 consecutive min of 0 activity counts and at most 2 min with counts between 0 and 100 [33,40,41,42,43,44]. Alcohol consumption had four categories: non-drinker, moderate drinker, heavy drinker, and missing alcohol. The “missing alcohol” category was created because 86 study participants did not have the alcohol consumption information available. All-cause mortality was defined for each time horizon between one and five years. For example, the three-year all-cause mortality was defined as study participants who died in the first three years after accelerometry data collection. Thus, all mortality cases for one-year all-cause mortality were included among the cases for three-year all-cause mortality. Table 1 provides the demographic summaries separated by alive and deceased status for each of the one- to five-year all-cause mortality outcomes in the accelerometry study in NHANES 2003–2006 cohorts.

### 2.2. Variables Used in the Statistical Analysis

#### 2.2.1. Traditional Mortality Predictors

The demographic, socioeconomic and health related predictors used in the analysis are age, gender, race (White, Black, Mexican American, Other, Other Hispanic), education level (less than high school, high school, more than high school), smoking status (never, former, current), drinking status (non-drinker, moderate drinker, heavy drinker, missing alcohol), body mass index (BMI, kg/m^2^), mobility problem (any difficulty, no difficulty), diabetes (yes/no), coronary heart disease (CHD, yes/no), congestive heart failure (CHF, yes/no), stroke (yes/no), cancer (yes/no), systolic blood pressure (SYS, mmHg), total cholesterol (LBXTC, mg/dL), and HDL cholesterol (LBDHDD, mg/dL).

#### 2.2.2. Accelerometry Derived Predictors

The physical activity data were collected by a hip-worn ActiGraph AM-7164 accelerometer (formerly the CSA/MTI AM-7164 manufactured by ActiGraph of Ft. Walton Beach, FL, USA) that provided minute level activity counts, a proprietary summary of accelerometry, for a period of seven days. These data were further summarized at the day level and the resulting summaries were averaged over valid days. For the analyses in this paper the following accelerometry derived predictors were used: (1) total activity count (TAC), the sum of minute level activity counts; (2) total log activity count (TLAC), the sum of log(1+activity count) at the minute level; (3) total minutes of moderate to vigorous physical activity (MVPA); (4) total minutes of light intensity physical activity (LIPA); (5) sedentary to active transition probability (SATP), the ratio of the total number of sedentary bouts to the total sedentary time; (6) active to sedentary transition probability (ASTP), the ratio of the total number of active bouts to the total activity time [45]; (7) total minutes of wear time (WT); and (8) total minutes of sedentary time (ST). A sedentary minute was defined as having an activity count smaller than 100, an active minute was defined as having an activity count larger or equal to 100, a LIPA minute was defined as having an activity count greater or equal to 100 and less than 2020, and an MVPA minute was defined as having an activity count greater or equal to 2020 [33,45]. Thus, the sum of ST, LIPA, and MVPA minutes is equal to 1440, the number of minutes in a day. Due to the deterministic association between ST, LIPA, and MVPA, only two of the variables are included in any given model.

Additional summary measures were obtained using functional principal component analysis (fPCA) of the daily activity trajectories. The fPCA decompositions were obtained using the fpca.face() function in the refund package [46] in R [47]. The procedure was applied to the data matrix where each row corresponds to a subject/day activity profile of the log (1+activity count) data and every column corresponds to a particular time of the day. The first six principal components explained approximately 57% of the variability in the activity data. Principal component scores were obtained for each wear day and each of the first six principal components and were summarized at the study participant level by taking the mean and standard deviations over the individual specific scores. This resulted in 12 additional summaries (mean and standard deviations of scores for six principal components). A backward selection approach was used on these twelve summaries separately for each mortality prediction horizon (one to five years). The criterion used for backward selection was the efficient parsimony information criteria (EPIC) [48]. For the one- and two-year all-cause mortality prediction the mean of the first principal component scores was significant. For three-, four-, and five-year mortality the mean of the first and of the second principal component scores and the standard deviation of the sixth principal component scores were statistically significant. The mean of the fourth principal component scores was significant in the four-year all-cause mortality model, while the standard deviation of the fifth principal component scores was significant in the five-year all-cause mortality model. This procedure was used for screening variables derived from fPCA decompositions and did not account for traditional or accelerometry-derived physical activity predictors. To facilitate interpretability of the PCA measures we replaced them with surrogate variables that can be calculated directly from the data [49].

### 2.3. Statistical Analysis

To account for the complex survey design of NHANES, participant demographic characteristics were obtained using the tableone package [50] in R. For each prediction horizon univariate (one predictor at a time) survey weighted logistic regression was conducted to compare the individual prediction performance. Predictors were ranked in decreasing order of the cross-validated area under the curve (CV-AUC) separately for each outcome for one- to five-year all-cause mortality. The ranking and prediction performance of the top seven predictors in the five-year all-cause mortality model were compared across the one- to five-year mortality models. A forward selection survey weighted multivariate logistic regression model was obtained for each mortality prediction horizon (one- to five-year) by minimizing EPIC [48] and the CV-AUC was reported for all resulting models. This resulted in five models, each corresponding to a different mortality prediction horizon. To compare the relative prediction performance of covariates across models, a combined model was obtained by selecting all significant predictors from these five models. This combined model was then fitted to each horizon data to quantify changes in estimated coefficients across prediction horizons. CV-AUC was calculated for the combined model using each year mortality data. In each horizon model, the variable predictive importance was evaluated as the difference between CV-AUC in the full union model and the combined model without that variable. All survey weighted logistic regression models were estimated using the svyglm() function in the R package survey [51].

## 3. Results

Table 1 provides the unadjusted number of alive and deceased participants and complex-survey adjusted participant characteristics by mortality status for all prediction time horizons (one- to five-year all-cause mortality datasets). The study participants for all models were the same (sample size 2978), though the subset of study participants who were alive/deceased changed with the prediction time horizon. For example, there were 2932 study participants who were alive after one year and 2681 who were alive after five years from the time of accelerometry data collection. The mean age varied slightly for the alive groups between 62.5 and 63.07 years and for the deceased groups between 71.81 and 73.23 years in the one-year and five-year mortality datasets, respectively. The percent of men who were alive decreased slightly from 46.6% in the one-year and two-year mortality datasets to 45.9% in the four-year and five-year mortality datasets. Correspondingly, the percent of women who were alive increased slightly from 53.4% in the one-year and two-year mortality datasets to 54.1% in the four-year and five-year mortality datasets. The percentage of men who died was higher for all prediction horizons and varied between 51.2% for the two-year mortality and 60.4% for the four-year mortality datasets. It is possible to have a small decrease in the percentage of men in the alive group and a larger proportion of men who died in each time horizon because many more study participants were alive in all time horizons.

The percent of alive participants varied slightly within race groups across prediction time horizons. For example, the percent of alive white participants varied between 79.9% and 80.2% in the one-year to five-year mortality datasets. The percent of alive black study participants varied only slightly around the average 9.4% for all time horizons. There are larger proportions of white participants in the alive and deceased groups because there is a larger proportion of white study participants. Among white study participants the proportion of alive is higher than that of deceased study participants in the one-year (80.1% alive and 79.8% deceased) and three-year horizons (80.2% alive and 78.5% deceased).

The percent of alive participants varied slightly within BMI groups across prediction time horizons. The percent of alive and overweight participants varied between 38.2% and 38.6% in the one-year and five-year mortality data sets, respectively. The percent of alive and obese participants was around 34.1% across horizons. Among study participants with normal BMI, the proportion of alive is lower than that of deceased study participants across all time horizons. Among the overweight study participants, the proportion of alive is higher than that of deceased study participants across all time horizons. For the obese category, the proportion of alive is lower than that of deceased study participants after one year and up to four years, while the opposite holds for the five-year time horizon.

The percent of alive participants varied slightly within education groups across prediction time horizons. For example, the percent of alive study participants who have less than a high school education level varied between 18.9% and 18% in the one-year and five-year mortality data sets. The percent of alive study participants with a high school degree remained roughly the same around 26.8 to 26.9% across all time horizons. The percent of alive participants who have a higher than high school education degree varied between and 54.2% and 55.2% in the one-year and five-year mortality data sets, respectively. Among these participants the proportion of alive subjects was higher than that of deceased study participants.

Participant survey-adjusted comorbidities and physical activity by mortality status for each prediction horizon are reported in Table A1 (Appendix A) of the appendix materials. Participants who died within each horizon had less time in moderate to vigorous activity (MVPA) except for the two-year horizon, higher active to sedentary transition probability (ASTP), and lower total activity and log activity counts. There was a smaller proportion of moderate drinkers, and a larger proportion of non-drinkers and heavy drinkers in the deceased group compared to the alive group across all prediction horizons. The proportion of participants with CHF, CHD and diabetes was consistently higher among those who died across all prediction horizons.

**Figure 1:**
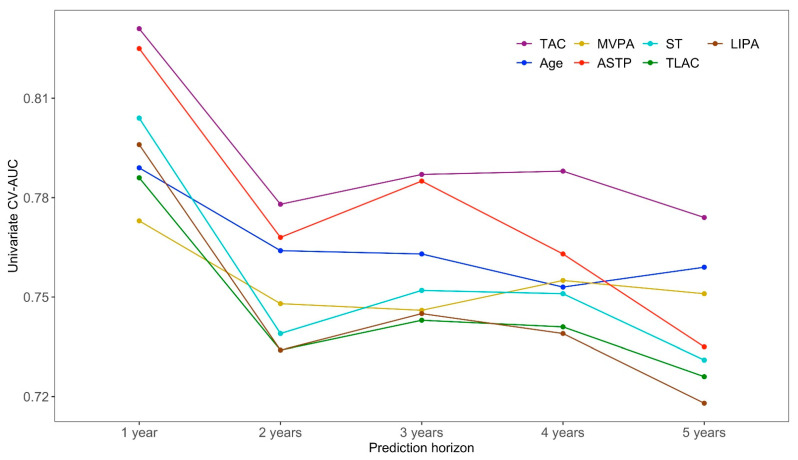
The cross-validated area under the curve (CV-AUC) values for the top seven predictors in the univariate five-year all-cause mortality logistic regression models across one- to five-year mortality prediction horizons. The x-axis corresponds to the prediction horizon and the y-axis is the CV-AUC (higher indicates better prediction performance).

Figure 1 displays the CV-AUC for the top seven predictors in the five-year univariate mortality model across each prediction horizon (one- to five-year all-cause mortality). All predictors had large CV-AUC across all prediction horizons (note the y-axis scale) and included six accelerometry-derived physical activity measures (TAC, MVPA, ASTP, ST, TLAC, and LIPA) and age. The total activity count (TAC, shown in violet in Figure 1) was consistently the top-ranking predictor in all prediction horizons with the corresponding CV-AUC decreasing from 0.831 in the one-year all-cause mortality model to 0.774 in the five-year all-cause mortality model. The second highest ranking predictor in the five-year mortality model was age (shown in dark blue in Figure 1) with CV-AUC 0.759, though it was outperformed in some time horizons by ASTP (one- to four-year), sedentary time and LIPA (one-year mortality), and MVPA (four-year mortality). The third through seventh ranking univariate five-year mortality predictors were the accelerometry-derived physical activity measures MVPA, ASTP, ST, TLAC, and LIPA, respectively. The rankings of these predictors changed slightly across prediction horizons, though they always stayed among the top seven predictors of mortality. Similar CV-AUC results are provided for the other, lower ranked, traditional predictors and accelerometry-derived physical activity measures in Table A2 of the appendix materials.

**Figure A1:**
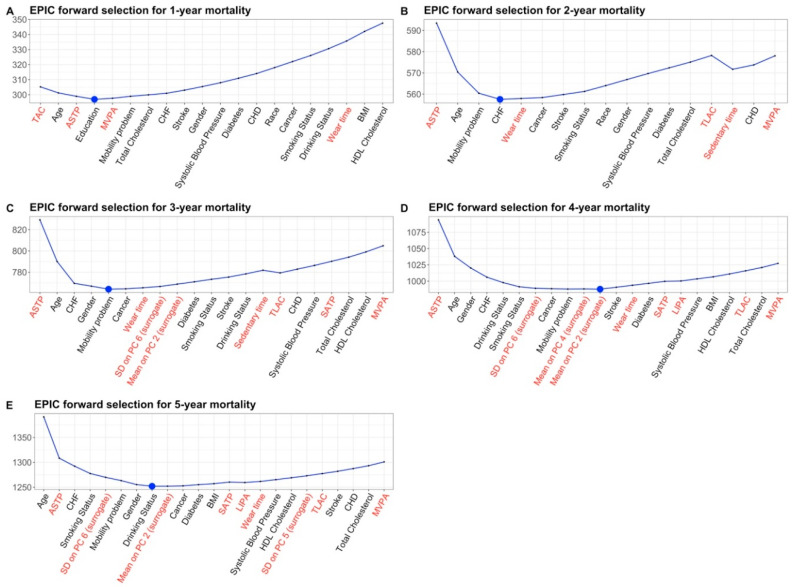
Multivariate models for one- to five-year all-cause mortality plotted as a function of the forward selection procedure. Accelerometry predictors are shown in red on the horizontal ax-is. NHANES Pooled Cohorts Study, United States, 2003–2006.

Models obtained using forward selection for each year prediction horizon are shown in Table 2 and in the Figure A1 of the appendix materials. The one-year all-cause mortality prediction model included four variables, TAC, age, ASTP, and education. The two-year prediction model included four variables, ASTP, age, mobility problem, and CHF. The three-year prediction model included five variables, ASTP, age, CHF, gender, and mobility problem. The four-year prediction model included 11 variables: ASTP, age, gender, CHF, drinking status, smoking status, the surrogate for the standard deviation on PC6, cancer, mobility problem, the surrogate for the mean on PC4, and the surrogate for the mean on PC2. Finally, the five-year horizon model included eight variables, age, ASTP, CHF, smoking status, the surrogate for the SD on PC 6, mobility problem, gender, and drinking status. For all years, variables are listed in the order they entered the model, which partially reflects their relative prediction performance. The models for shorter time mortality prediction (one- to three-year) tended to select a smaller number of variables because the number of events is smaller and thus, have less power to detect significant associations. All models included age and one or multiple accelerometry-derived physical activity measures with TAC and ASTP being among the strongest predictors. A combined prediction model for all five time horizons was obtained by selecting significant predictors across all individual time horizon models. This combined model included age, ASTP, mobility problem, CHF, gender, drinking and smoking status, the surrogate for the SD on PC 6, cancer, and the surrogate for the mean on PC 2. The CV-AUC for the forward selection and combined models were consistently high: (1) 0.883 and 0.843 for the forward selection and combined model for one-year all-cause mortality, respectively; (2) 0.829 and 0.841 for the forward selection and combined model for two-year all-cause mortality, respectively; (3) 0.845 and 0.852 for the forward selection and combined model for three-year all-cause mortality, respectively; (4) 0.852 and 0.853 for the forward selection and combined model for three-year all-cause mortality, respectively; and (5) 0.839 and 0.837 for the forward selection and combined model for five-year all-cause mortality.

The combined model performs consistently well and has the advantage that it replaces five models with one. Moreover, using the combined model allows a direct comparison of the relative prediction performance of variables across time horizons.

**Figure 2:**
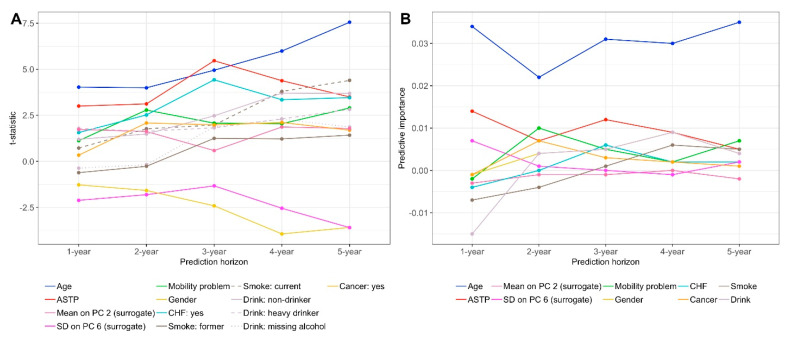
(**A**) The t-statistic values of the combined model coefficients in each prediction horizon. Each line corresponds to a t-statistic coefficient value (y-axis) as a function of the horizon prediction model (x-axis). (**B**) Predictive importance of each variable in the combined model measured by the difference between the CV-AUC of the full combined model and the combined model without that variable across prediction horizons.

Panel A in Figure 2 displays the t-statistic values for all variables in the combined model across years. Larger absolute values of the t-statistic correspond to stronger statistical evidence against the null hypothesis that a particular variable is not associated with mortality. Age, shown in blue with t-statistics values approximately in the range of 4 to 6, and ASTP, shown in red with t-statistics approximately in the range of 3 to 5, have the largest positive values of the t-statistic across years one through four. For year 5 the t-statistic for drinking is larger than for ASTP, though the t-statistic for ASTP is still large (3.497). This indicates that older individuals with more frequent transitions from active to sedentary activities have a higher mortality risk. Coronary heart failure (CHF), shown in light blue, has consistently high t-statistics values in years 2 through 5 (values between 2.519 for year 2 and 3.462 for year 5). These t-statistics are also the highest among variables quantifying comorbidities. This indicates that CHF is among the most predictive comorbidities for mortality across multiple time horizons. The smaller t-statistic for CHF for year one is likely due to the low number of study participants who died in the first year and had CHF at baseline (n = 9); for more details, see Table A3 in the Appendix. Gender has the smallest negative t-statistics (large in absolute value) in years three through five (–2.418, –3.946, and –3.596) indicating that being a female is statistically associated with a reduced risk of mortality in these time horizons. Similarly, the surrogate for the standard deviation of the sixth principal component, has the smallest negative t-statistics (large in absolute value) in years one and two. The surrogate for the standard deviation of the sixth principal component was computed as the standard deviation of the difference in average log-transformed activity counts comparing the time intervals (8 a.m.–10 a.m., 3 p.m.–5 p.m., 10 p.m.–12 a.m.) to (5 a.m.–7 a.m., 11 a.m.–1 p.m., 6 p.m.–8 p.m.) [34].

A limitation of t-statistics is that they depend strongly on the sample size. Indeed, with larger sample sizes and number of cases the t-statistics are likely to increase substantially due to the reduction in standard error. Therefore, for each variable and time horizons we investigated the effect on CV-AUC of adding that particular variable to all other variables in the combined model. Panel B in Figure 2 displays the variable relative prediction performance as measured by the differences between the CV-AUC in the combined model and the CV-AUC in the combined model without that variable. Results are consistent with the prediction performance measured by the t-statistics, at least in terms of ordering of variables. According to the improvement in CV-AUC in the combined model criterion, age has the strongest relative prediction performance across all years (difference in CV-AUC between 0.025 and 0.035). ASTP is second with an improvement in CV-AUC around 0.01. For the other predictors, the relative prediction performance varies across mortality prediction horizons. Indeed, some variables actually decrease the prediction performance of the model for certain years. This could be due to over-fitting the data and to inherent sampling variability.

## 4. Discussion

There is a rich literature indicating that objective PA features obtained from wearable accelerometers are strong predictors of all-cause mortality. We hypothesized that physical activity features would be highly predictive of all-cause mortality in short and medium follow-up periods. The current study supports our hypothesis by showing that objective physical activity measures outperform traditional mortality risk factors in adults over 50 years old from one to five years after the baseline measurement. We developed optimal prediction models for each individual time horizon as well as a combined model for all time horizons. The reasons for developing the combined model were (1) to simplify the number of models and increase the translational component of the paper; (2) to compare the association measures (e.g., covariate-specific t-statistics) and prediction measures (e.g., leave-one-covariate out loss in cross validated AUC) across time horizons; and (3) to build a reference all-cause mortality model that contained both traditional and objective physical activity risk factors. The prediction performance (as measured by cross-validated AUC) of the combined model was comparable to that of individual optimal models developed for the horizon-specific models, with some minimal losses, likely due to over-fitting in the shorter time horizons. 

Conducting analyses for multiple time horizons provides complementary information to previous studies that have either focused on one-time horizon [9] or on time-to-event analyses [37]. Single time horizons (e.g., five- or ten-year all-cause mortality) could miss important differences in predictive performance. These models further allow to investigate the hypothesis that the predictive value of PA features is most strong over shorted follow up periods, but persists when looking at longer follow up times. In single variable models we found that objective physical activity measurements strongly outperform age in shorter time horizons (one to three years all-cause mortality). In longer time horizons (four to five years all-cause mortality) these differences are reduced and can change sign (that is, age can become a stronger predictor). Standard time-to-event analyses are not designed and have not been used to quantify the different effects of risk factors on mortality by prediction horizon. They could be modified to do that using interactions between time and covariates, but we consider that the separate analysis for individual mortality time horizons: (1) is easier to interpret and visualize; (2) provides insights into the types of interactions that could be explored in the future; and (3) avoids some of the assumptions of time-to-event analyses (e.g., proportional hazards). The current study examines immediate (one-year prediction horizon) to medium term (five-year prediction horizon) effects of objective measures of physical activity on mortality prediction. Results indicate that the statistical evidence (measured by the t-statistic in the combined model) against the null hypothesis that age is not associated with mortality increases with the prediction horizon (from around 4.03 for year 1 to 7.56 for year 5). In the combined model the active to sedentary transition probability (ASTP) is the second strongest predictor of mortality with t-statistics between 3.00 for year 1 and 3.50 for year 5; see Panel A in Figure 2. These results complement the results from single variable regressions (see Figure 1), where the total activity count (TAC) outperformed both age and ASTP in all time horizons.

An important finding of this study is that we built a combined prediction model for all five time horizons. This combined model includes age, ASTP, mobility problem, CHF, gender, drinking, smoking, the surrogate for the SD on PC 6, cancer, and the surrogate for the mean on the PC 2. The prediction performance of the combined model and the optimal horizon-specific models were consistently high: (1) for the one-year all-cause mortality CV-AUC was 0.843 for the combined and 0.883 for the optimal model; (2) for the two-year all-cause mortality CV-AUC was 0.829 for the combined and 0.841 for the optimal model; (3) for the three-year all-cause mortality CV-AUC was 0.845 for the combined and 0.852 for the optimal model; (4) for the three-year all-cause mortality CV-AUC was 0.852 for the combined and 0.853 for the optimal model; and (5) for the three-year all-cause mortality CV-AUC was 0.837 for the combined and 0.839 for the optimal model.

The current study has several limitations. First, only main effects were used, and interaction terms were not considered to preserve simplicity and to allow for clearer interpretation of the results. Second, there are fewer deaths in the shorter time horizons, which leads to more variable estimates that, in turn, may lead to less stable and generalizable results. Third, the combined model has more predictors than the optimal models in shorter horizons, which leads to some loss in predictive performance, likely due to overfitting. Fourth, we concentrated on short prediction horizons even though the maximum available follow up time for this study was 13.083 years. This was done to study the association between objective physical activity measures and immediate to mid-term mortality. Studying the long-term effects in larger prediction horizons and potential interactions between physical activity measurements and time is a topic for future research.

The current study is not intended to address the question of causation; that is, we do not hypothesize that the level (high or low) of physical activity as measured by wearable devices is related to potential causes of death for older adults suffering from different comorbidity conditions. Rather, we propose (and statistically verify) the possibility of using PA as an additional diagnostic tool which may indicate higher or lower probability of death due to any cause one, two, three, four and five years after PA assessment using wearable accelerometry devices. An important limitation of using wearable accelerometers in PA assessment, is that these devices provide an unbiased estimate of ambulation, however stationary physical activity (e.g., weightlifting and core strength exercises) may not be accurately detected by the accelerometers. Nevertheless, since walking and similar types of activity are among the main sources of activity for most individuals, wearable accelerometry devices provide a reliable estimate of an overall daily PA level that is more complete and objective than self-reported information.

In summary, our conclusions support the results obtained in [9], which focused only on the five-year all-cause mortality for adults over 50 years of age in NHANES 2003–2006. Results also agree qualitatively with analyses based on the UK Biobank [37] study using time-to-event analysis. However, our results complement these studies, which have not parsed out results in individual time horizons. Indeed, focusing on different time horizons provides information that was previously unavailable (e.g., that the prediction performance of objective PA measurements is even better in shorter time horizons). Overall, we provide new insights into the prediction performance of objective measures of PA of mortality, an in-depth quantification of the prediction performance of objective measures of PA as a function of prediction horizon, and provide a single, combined model for assessing immediate to mid-term mortality risks.

## Figures and Tables

**Table 1:** Unadjusted number of alive and diseased participants and complex-survey adjusted demographic characteristics separated by alive and deceased status for 1-, 2-, 3-, 4-, 5-years after the participation in the accelerometry study in NHANES 2003–2006 cohorts. For each prediction horizon, the unadjusted number of participants is calculated as the total number of participants who were alive and deceased one, two, three, four and five years after participating in the study. The complex survey weighted number of participants in each category is calculated as the sum of all adjusted sampling weights for all participants in that category.

	Horizon 1		Horizon 2		Horizon 3		Horizon 4		Horizon 5	
	Alive (n = 2932)	Deceased (n = 46)	Alive (n = 2884)	Deceased (n = 94)	Alive (n = 2823)	Deceased (n = 155)	Alive (n = 2760)	Deceased (n = 218)	Alive (n = 2681)	Deceased (n = 297)
**Age**	63.07 (9.58)	73.23 (7.45)	62.96 (9.52)	72.29 (9.2)	62.82 (9.47)	72.23 (9.01)	62.69 (9.38)	71.86 (9.6)	62.5 (9.27)	71.81 (9.83)
**Gender:** Female	1572 (53.4%)	15.1 (48.1%)	1552.7 (53.4%)	34.4 (48.8%)	1537.2 (53.7%)	49.9 (44%)	1523.4 (54.1%)	63.8 (39.6%)	1492.4 (54.1%)	94.8 (43.2%)
**Gender:** Male	1374.5 (46.6%)	16.3 (51.9%)	1354.7 (46.6%)	36.2 (51.2%)	1327.2 (46.3%)	63.7 (56%)	1293.6 (45.9%)	97.3 (60.4%)	1266.3 (45.9%)	124.5 (56.8%)
**BMI:** Underweight	31.1 (1.1%)	1.7 (5.4%)	29.1 (1%)	3.7 (5.3%)	28.2 (1%)	4.7 (4.1%)	27.6 (1%)	5.3 (3.3%)	27.6 (1%)	5.3 (2.4%)
**BMI:** Normal	786.7 (26.7%)	9.3 (29.6%)	772.8 (26.6%)	23.2 (32.9%)	758 (26.5%)	38 (33.5%)	743 (26.4%)	53 (32.9%)	722.5 (26.2%)	73.6 (33.5%)
**BMI:** Overweight	1124.6 (38.2%)	7.9 (25.3%)	1113.1 (38.3%)	19.4 (27.5%)	1100.8 (38.4%)	31.8 (27.9%)	1084.9 (38.5%)	47.6 (29.6%)	1064.8 (38.6%)	67.7 (30.9%)
**BMI:** Obese	1004.2 (34.1%)	12.5 (39.7%)	992.4 (34.1%)	24.2 (34.3%)	977.5 (34.1%)	39.1 (34.4%)	961.5 (34.1%)	55.2 (34.3%)	943.9 (34.2%)	72.8 (33.2%)
**Race:** White	2361.1 (80.1%)	25.1 (79.8%)	2327.5 (80.1%)	58.6 (83%)	2296.9 (80.2%)	89.2 (78.5%)	2253.9 (80%)	132.2 (82.1%)	2205.4 (79.9%)	180.7 (82.4%)
**Race:** Mexican American	120.1 (4.1%)	0.5 (1.6%)	119.8 (4.1%)	0.8 (1.2%)	119.1 (4.2%)	1.5 (1.3%)	117.5 (4.2%)	3.1 (2%)	114.4 (4.1%)	6.2 (2.8%)
**Race:** Black	280 (9.5%)	4.4 (14.1%)	274.7 (9.4%)	9.8 (13.8%)	268.7 (9.4%)	15.8 (13.9%)	265.9 (9.4%)	18.5 (11.5%)	260.7 (9.4%)	23.8 (10.8%)
**Race:** Other	118.1 (4%)	1.4 (4.5%)	118.1 (4.1%)	1.4 (2%)	114.6 (4%)	4.9 (4.3%)	114.6 (4.1%)	4.9 (3.1%)	113.8 (4.1%)	5.7 (2.6%)
**Race:** Other Hispanic	67.3 (2.3%)	0 (0%)	67.3 (2.3%)	0 (0%)	65.1 (2.3%)	2.2 (1.9%)	65.1 (2.3%)	2.2 (1.4%)	64.4 (2.3%)	2.9 (1.3%)
**Education:** Less than high school	558.1 (18.9%)	8.1 (25.7%)	547 (18.8%)	19.2 (27.2%)	531.4 (18.6%)	34.8 (30.6%)	516 (18.3%)	50.2 (31.1%)	496.3 (18%)	69.9 (31.9%)
**Education:** High school	792.8 (26.9%)	16.8 (53.4%)	782.5 (26.9%)	27.2 (38.5%)	771.4 (26.9%)	38.5 (33.9%)	755.5 (26.8%)	54.1 (33.6%)	739.4 (26.8%)	70.2 (32%)
**Education:** More than high school	1595.6 (54.2%)	6.6 (20.9%)	1577.9 (54.3%)	24.2 (34.3%)	1561.8 (54.5%)	40.3 (35.5%)	1545.4 (54.9%)	56.8 (35.3%)	1523 (55.2%)	79.2 (36.1%)

**Table 2:** Forward regression selection results. Variables that were not selected in the specific horizon prediction model are left blank.

	Horizon 1	Horizon 2	Horizon 3	Horizon 4	Horizon 5
Predictor	Estimate (*p*-Value)	Estimate (*p*-Value)	Estimate (*p*-Value)	Estimate (*p*-Value)	Estimate (*p*-Value)
Age	0.057 (0.005)	0.061 (0.001)	0.066 (<0.001)	0.075 (<0.001)	0.083 (<0.001)
ASTP	0.494 (0.039)	0.569 (<0.001)	0.688 (<0.001)	0.511 (0.001)	0.404 (0.002)
Cancer: yes				0.426 (0.051)	
CHF: yes		0.861 (0.007)	1.158 (<0.001)	0.942 (0.003)	0.827 (0.003)
Heavy drinker				0.854 (0.040)	0.965 (0.013)
Missing alcohol				1.194 (0.034)	0.795 (0.080)
Non-drinker				0.780 (0.002)	0.603 (0.002)
Education: high-school	0.605 (0.163)				
Education: more than high-school	−0.717 (0.107)				
Gender: female			−0.608 (0.010)	−0.891 (0.001)	−0.640 (0.003)
Mobility problem		0.932 (0.005)	0.657 (0.026)	0.520 (0.050)	0.603 (0.009)
Mean on PC 2				0.153 (0.045)	
Mean on PC 4				−0.206 (0.062)	
Smoking: current				0.964 (0.001)	0.845 (< 0.001)
Smoking: former				0.245 (0.215)	0.340 (0.159)
SD on PC 6				−0.265 (0.015)	−0.277 (0.002)
TAC	−0.729 (0.307)				

## Appendix A

**Table A1:** Unadjusted number of alive and diseased participants and complex-survey adjusted comorbidities and physical activity characteristics separated by alive and deceased status for 1-, 2-, 3-, 4-, 5-years after the participation in the accelerometry study in NHANES 2003–2006 cohorts.

	Horizon 1		Horizon 2		Horizon 3		Horizon 4		Horizon 5	
	Alive (n = 2932)	Deceased (n = 46)	Alive (n = 2884)	Deceased (n = 94)	Alive (n = 2823)	Deceased (n = 155)	Alive (n = 2760)	Deceased (n = 218)	Alive (n = 2681)	Deceased (n = 297)
TAC	224,135.26 (114,718.3)	106,537.81 (72,247.73)	225,055.33 (114,023.62)	133,889.57 (119,354.91)	226,715.22 (113,748.63)	126,561.6 (103,550.69)	228,269.57 (113,468.66)	128,865.11 (99,724.72)	229,616.63 (113,240.73)	138,316.77 (102,558.28)
MVPA	15.72 (17.97)	5.5 (11.36)	15.79 (17.9)	8.26 (18.33)	15.95 (17.95)	7.04 (15.39)	16.13 (18.01)	6.63 (13.88)	16.31 (18.08)	6.9 (13.35)
ASTP	0.29 (0.08)	0.41 (0.11)	0.29 (0.08)	0.39 (0.12)	0.29 (0.08)	0.4 (0.11)	0.29 (0.08)	0.38 (0.11)	0.29 (0.08)	0.37 (0.11)
Sedentary Time	1100.69 (104.86)	1223.78 (105.61)	1099.73 (104.2)	1194.95 (120.86)	1098.1 (103.48)	1200.06 (111.54)	1096.63 (102.55)	1195.65 (114.22)	1095.5 (102.06)	1183.58 (115.14)
TLAC	2832.74 (703.73)	1987.89 (770.79)	2838.99 (699.39)	2199.09 (840.54)	2849.34 (695.33)	2180.43 (764.16)	2859 (688.22)	2208.48 (793.74)	2866.94 (684.91)	2281.36 (788.63)
LIPA	323.59 (97.55)	210.72 (101.45)	324.48 (97)	236.79 (111.05)	325.95 (96.33)	232.9 (104.14)	327.24 (95.42)	237.72 (108.38)	328.19 (94.99)	249.52 (109.12)
SATP	0.08 (0.02)	0.07 (0.02)	0.08 (0.02)	0.07 (0.02)	0.08 (0.02)	0.07 (0.02)	0.08 (0.02)	0.07 (0.02)	0.08 (0.02)	0.07 (0.02)
Total Cholesterol	206.63 (41.29)	202.87 (52.83)	206.94 (41.29)	191.93 (44.2)	207.25 (41.18)	189.84 (44.09)	207.33 (41.05)	193.56 (45.59)	207.57 (40.89)	194.26 (45.92)
CHF: yes	136 (4.6%)	6.6 (20.9%)	127.6 (4.4%)	15.1 (21.3%)	115.2 (4%)	27.4 (24.1%)	109.7 (3.9%)	32.9 (20.4%)	103.9 (3.8%)	38.7 (17.6%)
CHF: no	2180.5 (95.4%)	24.9 (79.1%)	2779.8 (95.6%)	55.6 (78.7%)	2749.2 (96%)	86.2 (75.9%)	2707.2 (96.1%)	128.2 (79.6%)	2654.8 (96.2%)	180.6 (82.4%)
Cancer: yes	470 (15.9%)	8.1 (25.8%)	455.4 (15.7%)	22.6 (32%)	444.3 (15.5%)	33.8 (29.7%)	431.3 (15.3%)	46.8 (29%)	418.3 (15.2%)	59.7 (27.2%)
Cancer: no	2476.6 (84.1%)	23.3 (74.2%)	2452 (84.3%)	48 (68%)	2420.1 (84.5%)	79.9 (70.3%)	2385.6 (84.7%)	114.3 (71%)	2340.4 (84.8%)	159.6 (72.8%)
Diabetes: yes	392.2 (13.3%)	8.3 (26.5%)	386.6 (13.3%)	13.9 (19.6%)	371.9 (13%)	28.6 (25.2%)	362.2 (12.9%)	38.3 (23.8%)	347.8 (12.6%)	52.7 (24%)
Diabetes: no	2554.4 (86.7%)	23.1 (73.5%)	2520.7 (86.7%)	56.7 (80.4%)	2492.5 (87%)	85 (74.8%)	2454.7 (87.1%)	122.8 (76.2%)	2410.9 (87.4%)	166.6 (76%)
CHD: yes	218.4 (7.4%)	4.8 (15.4%)	208.6 (7.2%)	14.7 (20.8%)	200.7 (7%)	22.6 (19.9%)	194.6 (6.9%)	28.7 (17.8%)	186 (6.7%)	37.3 (17%)
CHD: no	2728.1 (92.6%)	26.6 (84.6%)	2698.8 (92.8%)	55.9 (79.2%)	2663.7 (93%)	91 (80.1%)	2622.3 (93.1%)	132.4 (82.2%)	2572.7 (93.3%)	182 (83%)
Systolic Blood Pres	131.14 (20.3%)	141.2 (33.12%)	131.11 (20.17)	136.7 (30.79)	131.11 (20.18)	134.51 (27.21)	131 (20.18)	135.41 (25.16)	130.9 (20.04)	135.59 (25.24)
Stroke: yes	134.1 (4.6%)	3 (9.4%)	129.7 (4.5%)	7.4 (10.5%)	124.5 (4.3%)	12.6 (11.1%)	119.2 (4.2%)	17.9 (11.1%)	108.3 (3.9%)	28.8 (13.1%)
Stroke: no	2812.4 (95.4%)	28.5 (90.6%)	2777.7 (95.5%)	63.2 (89.5%)	2739.9 (95.7%)	101 (88.9%)	2697.8 (95.8%)	143.1 (88.9%)	2650.4 (96.1%)	190.5 (86.9%)
HDL Cholesterol	56.08 (16.47)	52.39 (21.86)	56.1 (16.46)	53.92 (19.36)	56.12 (16.47)	54.25 (18.02)	56.12 (16.4)	54.78 (18.8)	56.13 (16.43)	54.98 (17.86)
Wear Time	879.48 (125.1)	884.27 (168.04)	878.94 (124.55)	903.81 (162.16)	878.72 (124.17)	899.84 (156.72)	878.93 (123.95)	890.09 (151.58)	879.13 (122.83)	884.62 (156.53)
Mobility Problem:										
Any Difficulty	776.8 (26.4%)	21.1 (67.3%)	749.1 (25.8%)	48.9 (69.2%)	725.1 (25.3%)	72.9 (64.1%)	701.3 (24.9%)	96.7 (60%)	669.7 (24.3%)	128.3 (58.5%)
No Difficulty	2169.7 (73.6%)	10.3 (32.7%)	2158.3 (74.2%)	21.7 (30.8%)	2139.3 (74.7%)	40.8 (35.9%)	2115.7 (75.1%)	64.3 (40%)	2089 (75.7%)	91 (41.5%)
Drinking Status:										
Moderate Drinker	1559.9 (52.9%)	8.8 (27.9%)	1546.7 (53.2%)	22 (31.1%)	1535.8 (53.6%)	32.9 (28.9%)	1522.8 (54.1%)	45.8 (28.4%)	1500.2 (54.4%)	68.4 (31.2%)
Non-Drinker	1090.3 (37%)	18.3 (58.1%)	1068.2 (36.7%)	40.4 (57.2%)	1044 (36.4%)	64.6 (56.8%)	1016.7 (36.1%)	91.8 (57%)	990 (35.9%)	118.6 (54.1%)
Heavy Drinker	210.5 (7.1%)	4 (12.7%)	207.6 (7.1%)	6.9 (9.8%)	204.2 (7.1%)	10.3 (9.1%)	198.7 (7.1%)	15.8 (9.8%)	190.4 (6.9%)	24.1 (11%)
Missing alcohol	85.9 (2.9%)	0.4 (1.3%)	84.9 (2.9%)	1.4 (1.9%)	80.4 (2.8%)	5.9 (5.2%)	78.6 (2.8%)	7.6 (4.7%)	78.1 (2.8%)	8.1 (3.7%)
Smoking Status:										
Never	1350.6 (45.8%)	11.8 (37.4%)	1338 (46%)	24.4 (34.6%)	1327.4 (46.3%)	35 (30.8%)	1314.3 (46.7%)	48.1 (29.9%)	1295.8 (47%)	66.6 (30.4%)
Former	1093.1 (37.1%)	11.9 (37.9%)	1078.2 (37.1%)	26.9 (38%)	1054.6 (36.8%)	50.4 (44.4%)	1034.1 (36.7%)	70.9 (44%)	1003.9 (36.4%)	101.1 (46.1%)
Current	502.8 (17.1%)	7.8 (24.7%)	491.2 (16.9%)	19.4 (27.4%)	482.4 (16.8%)	28.2 (24.8%)	468.6 (16.6%)	42 (26.1%)	459 (16.6%)	51.6 (23.5%)

**Table A2:** Univariate cross-validated AUC for each prediction horizon. The variables are ranked according to year 5 horizon.

CV-AUC and Ranking	Horizon 1		Horizon 2		Horizon 3		Horizon 4		Horizon 5	
	AUC	Rank	AUC	Rank	AUC	Rank	AUC	Rank	AUC	Rank
TAC	0.831	1	0.778	1	0.787	1	0.788	1	0.774	1
Age	0.789	5	0.764	3	0.763	3	0.753	4	0.759	2
MVPA	0.773	7	0.748	4	0.746	5	0.755	3	0.751	3
ASTP	0.825	2	0.768	2	0.785	2	0.763	2	0.735	4
Sedentary time	0.804	3	0.739	5	0.752	4	0.751	5	0.731	5
TLAC	0.786	6	0.734	6	0.743	7	0.741	6	0.726	6
LIPA	0.796	4	0.734	7	0.745	6	0.739	7	0.718	7
Mobility problem	0.713	8	0.718	8	0.691	8	0.677	8	0.676	8
SD on PC 6					0.663	9	0.665	10	0.663	9
SATP	0.700	9	0.663	9	0.652	10	0.669	9	0.662	10
SD on PC 5									0.623	11
Education	0.673	10	0.566	16	0.602	13	0.609	12	0.610	12
Drinking status	0.607	12	0.604	11	0.618	12	0.619	11	0.598	13
Total cholesterol	0.474	23	0.619	10	0.622	11	0.602	13	0.593	14
Smoking status	0.528	20	0.572	14	0.568	17	0.594	14	0.590	15
CHF	0.584	14	0.588	12	0.597	14	0.583	15	0.569	16
Cancer	0.540	16	0.577	13	0.572	15	0.570	17	0.561	17
BMI	0.501	22	0.559	18	0.572	16	0.563	19	0.559	18
Diabetes	0.563	15	0.532	22	0.558	19	0.554	21	0.557	19
Gender	0.535	17	0.523	23	0.544	20	0.573	16	0.556	20
CHD	0.528	19	0.561	17	0.561	18	0.554	20	0.550	21
Systolic blood pressure	0.584	13	0.534	20	0.523	23	0.548	22	0.549	22
Stroke	0.531	18	0.533	21	0.533	22	0.537	24	0.545	23
HDL cholesterol	0.620	11	0.568	15	0.539	21	0.543	23	0.535	24
Race	0.512	21	0.555	19	0.517	24	0.528	25	0.523	25
Wear time	0.416	24	0.519	24	0.517	25	0.497	27	0.472	26
Mean on PC 2					0.454	26	0.511	26	0.434	27
Mean on PC 4							0.567	18		

**Table A3:** t-statistic values of the variables selected in the combined model across years.

Variable	Horizon 1	Horizon 2	Horizon 3	Horizon 4	Horizon 5
Age	4.030	3.994	4.950	5.990	7.555
ASTP	3.001	3.118	5.466	4.380	3.497
Cancer: yes	0.334	2.083	1.980	2.109	1.693
CHF: yes	1.563	2.519	4.428	3.344	3.462
Heavy drinker	1.786	1.632	1.820	2.304	2.822
Missing alcohol	−0.373	−0.187	1.873	2.287	1.865
Non-drinker	1.195	1.484	2.473	3.703	3.685
Gender	−1.278	−1.584	−2.418	−3.946	−3.596
Mean on PC 2	1.726	1.619	0.583	1.865	1.782
Mobility problem	1.121	2.787	2.072	2.042	2.897
SD on PC 6	−2.120	−1.813	−1.334	−2.552	−3.603
Smoke: current	0.720	1.765	1.971	3.797	4.395
Smoke: former	−0.617	−0.272	1.248	1.217	1.421
